# Tomographic Findings in the Retina of Unvaccinated Patients with COVID Pneumonia: Prospective Longitudinal Study

**DOI:** 10.3390/ijerph20095659

**Published:** 2023-04-27

**Authors:** Carlos Enrique Monera Lucas, Manuel Vicente Baeza Diaz, Jose A. Quesada, Adriana Lopez-Pineda, Cristian Fernandez Martinez, Jose Juan Martinez Toldos, Vicente F. Gil-Guillén

**Affiliations:** 1 Retina Unit, Ophthalmology Service, General University Hospital of Elche, 03203 Elche, Spain; 2 Clinical Medicine Department, Miguel Hernandez University, 03550 San Juan de Alicante, Spain; 3 Network for Research on Chronicity, Primary Care, and Health Promotion (RICAPPS), 03550 Alicante, Spain; 4 Department of Pathology and Surgery, Miguel Hernandez University, 03550 San Juan de Alicante, Spain

**Keywords:** eye, retina, COVID-19, coronavirus infection, pneumonia, tomography, X-ray computed

## Abstract

There is no definitive evidence on the extent of SARS-CoV-2’s effect on the retina. This study aims to determine if the natural history of SARS-CoV-2 infection affects tomographic findings in the retina of patients with COVID-19 pneumonia. This is a prospective cohort study of patients hospitalized with COVID-19 pneumonia. The patients underwent ophthalmological explorations and optical coherence tomography during the acute phase of the infection and at a follow-up 12 weeks later. The primary outcomes were the central retinal thickness and central choroidal thickness, which were compared longitudinally and with non-COVID-19 historical controls. No statistically relevant differences were observed in the longitudinal analysis of the thickness of the central retina (*p* = 0.056), central choroid (*p* = 0.99), retinal nerve fiber layer (*p* = 0.21), or ganglion cell layer (*p* = 0.32). Patients with acute COVID-19 pneumonia showed significantly greater central retinal thickness than non-COVID controls (*p* = 0.006). In conclusion, tomographic measures of the retina and choroid are not influenced by the phase of COVID-19 infection and remain stable during 12 weeks. The central retinal thickness may increase in the acute phase of COVID-19 pneumonia, but more epidemiological studies using optical coherence tomography in the early stages of the disease are needed.

## 1. Introduction

The determinants of the severity of the clinical course of COVID-19 are not completely clear, although ongoing research is exploring new hypotheses related to the involvement of different inflammatory cytokines, which may have an important role in the pathophysiology of the disease [1]. In addition, there have been reports of coagulation disorders [2] in patients with COVID-19 and of thromboembolic events [3,4] in people with a history of the disease, with some authors raising the possibility of a cytopathic effect of the virus on the vascular endothelium and other cell groups [5]. Although most patients with COVID-19 experience respiratory symptoms, the disease is systemic, encompassing a very broad clinical spectrum including gastrointestinal [6], neurological [7], skin [8], and ophthalmological symptoms [9].

At the ocular level, COVID-19 mainly causes surface symptoms and conjunctivitis, and the presence of SARS-CoV-2 has been detected in the tear film. Some cases of thrombotic events in the great vessels of the retina, along with findings of inflammatory signs and exudates in the posterior segment, have also been reported [10,11]. Studies using retinal optical coherence tomography (OCT) tests in patients with COVID-19 are inconclusive, as they are influenced by both the clinical phase of the disease and by patient selection. However, knowing whether there are clinical or tomographic impacts on the retina during the acute phase of COVID-19 would help determine if the SARS-CoV-2 infection is a risk factor for vascular complications in the retina later on. Moreover, studying the choroid in these patients is of interest, since changes in the choroidal structure can affect the function of the blood-retinal barrier and the metabolism of the retinal pigment epithelium, giving rise to retinal pathologies with visual repercussions [12,13]. The determination of structural alterations in the retina and choroid of patients affected by COVID-19 could be useful for identifying patients at higher risk of long-term visual sequelae, and potentially for signaling a worse prognosis at the systemic level. This study aims to assess differences in the tomographic findings of the retina and choroid in patients with acute COVID pneumonia versus controls without COVID-19 and to evaluate if these differences are maintained in the recovery phase.

## 2. Materials and Methods

A cross-sectional study was performed to compare OCT findings in a sample of patients with acute COVID-19 pneumonia versus a group of non-COVID controls. The COVID-19 group was followed longitudinally and prospectively to compare the tomographic status of the retina in the acute phase (T0) versus the recovery phase (T1). The study took place between November 2020 and April 2021 at the Elche General University Hospital (Spain).

### 2.1. Study Population

The patients included were adults with an active SARS-CoV-2 infection, confirmed by polymerase chain reaction (PCR) or an antigen test, diagnosed with COVID pneumonia in the emergency department of the Elche General University Hospital, and admitted to the hospital’s infectious diseases unit. All participants signed their informed consent. The clinical severity of pneumonia and the indication for hospital admission were defined by the presence of any of the following symptoms: dyspnea, altered level of consciousness, hypoxemia or hypercapnia at rest, and/or arterial hypotension. The exclusion criteria were as follows: history of trauma or recent eye surgery (<3 months), clinical condition that impeded the performance of tests or collection of data (mobility problems, poor collaboration, or contraindication for sitting), and ocular comorbidity that could affect the outcome variables (refractive surgery, high myopia, diabetic retinopathy, maculopathy, and advanced glaucoma). Patients in the COVID-19 group were recruited by consecutive sampling from 24 November to 20 December 2020.

Non-COVID controls were drawn from a normative, historical database of OCT studies performed with the DRI Triton platform in adults from the same health department (Elche General University Hospital) as the patients in the COVID-19 group. Patient characteristics (age, sex, comorbidity) were comparable between groups. Controls were selected from a time period prior to the COVID pandemic to ensure that they had never been infected nor received the vaccine.

### 2.2. Data Collection and Measurement

Recruitment and follow-up were performed in all participants before the introduction of COVID-19 vaccines. The data from the COVID-19 group were collected at two time points: during hospital admission (T0, in the acute, contagious phase of the infection) and 12 weeks after hospital discharge (T1, in the late phase of the infection, once all PCR and antigen detection tests were negative).

Patients in the COVID-19 group were invited to carry out the tests and measurements necessary to obtain the study data. All tests were non-invasive, requiring the instillation of ocular mydriatics (tropicamide) 30 min beforehand. Since examining the patients during hospital admission represented a clear exposure risk for the researcher, all preventive and isolation measures recommended by health institutions were adopted (personal protective equipment, respiratory isolation, hand hygiene, and sterilization of the instruments before and after the examination). Because patients were isolated in their hospital rooms on the admission ward, and the testing platform and tools had to be moved to examine each participant.

**Figure 1:**
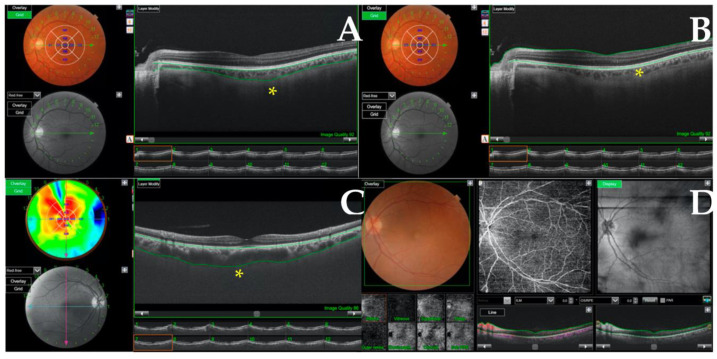
Optical coherence tomography (OCT) and OCT angiography performed in patients. (**A**,**B**) show an analysis of the choroidal thicknesses and central retina, respectively, marked with (*). (**C**) shows an increased choroidal thickness with morphological pachychoroid changes. (**D**) shows an OCT-angiography, which detects focal changes in blood flow density in the deep vascular plexuses of the retina and enables identification of potential neovascular lesions.

Patients underwent a fundus examination with an indirect ophthalmoscope. Using a DRI Triton device (Topcon Inc., Tokyo, Japan), macula and papilla retinography images were taken, and an OCT angiography was performed in the macular and peripapillary area using the “radial” image acquisition option (Figure 1). The thickness variables automatically generated by the platform software were obtained in the GRID option (Figure 1C). Using the GRID tool (arrow), we obtained the mean thicknesses of all the images in the analyzed layers (asterisk). Previously, the researchers had verified the correct identification and segmentation of the retinal layers, which the software usually does automatically.

### 2.3. Study Variables

In the COVID-19 group, the following variables were collected at baseline: age, sex, smoking (yes/no), and diagnosis of diabetes mellitus (yes/no) and hypertension (yes/no). Analytical variables from the blood tests performed upon admission were also recorded: plasma fibrinogen (mg/dL), D-dimers (µg/mL), and interleukin 6 (IL-6, pg/mL). The other variables were the eye evaluated (right/left), plus the intervals in days from the onset of symptoms to admission, diagnosis of the SARS-CoV-2 infection on admission, and admission to optical examinations. Finally, the following outcome variables were collected both in the acute phase (T0) and in the recovery phase (T1): presence (yes/no) of ischemic or inflammatory clinical signs in the posterior segment (retinal hemorrhage, vasculitis papilledema, central retinal artery occlusion, vitritis, cotton wool exudates, macular edema, or areas of retinal ischemia), central retinal thickness (CRT, µm), retinal nerve fiber layer (RNFL) thickness (µm), and ganglion cell layer (GCL) thickness (µm) and central choroid thickness (CCT, µm).

Variables collected in non-COVID-19 controls were age, sex, the eye examined tomographically (right/left), and the results of the tomography (CRT, RNFL, GCL, and CCT in µm). No other variables were recorded because they were not available in the database. The primary outcome variables were CRT and CCT; and the secondary variables were RNFL and GCL.

### 2.4. Statistical Analysis

The variables were entered into a database and statistically analyzed. In the descriptive analysis, qualitative variables were expressed as absolute frequencies and proportions, and quantitative ones as the mean and standard deviation (SD) or median and interquartile range (IQR), as appropriate. The normality of the distribution was verified with the Kolmogorov-Smirnov test.

To compare the tomographic findings between COVID-19 and non-COVID-19 patients, we used the measurements obtained on admission to hospital (T0) and from the most recent tomography study available, respectively. Homogeneity between groups was analyzed using the chi-squared test and Student’s *t* test. An analysis of covariance was performed for CRT and CCT, variables which were normally distributed, and the homogeneity of variances was evaluated using Levene’s test. RNFL and GCL did not have a normal distribution, so the non-parametric Mann-Whitney U test was used.

For the longitudinal analysis in COVID-19 patients, the mean values of CRT and CCT were compared at T0 and T1 using Student’s *t* test. A general linear model of repeated measures was fitted with between- and within-subject factors to limit the effect of the covariates age, fibrinogen, D-dimer, and IL-6. To analyze changes in RNFL and GCL (variables with non-normal distribution), the Wilcoxon non-parametric test was applied. Statistical analyses were performed using SPSS software (IBM Corp. SPSS Statistics for Windows, Version 28.0, 2021, Armonk, NY, USA: IBM Corp).

## 3. Results

**Figure 2:**
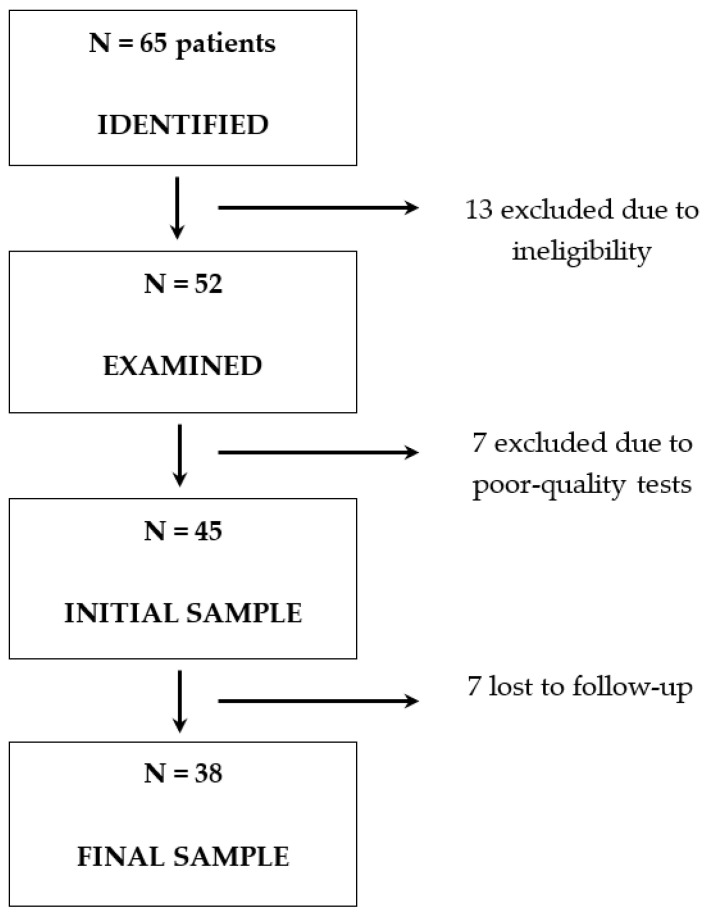
Patient flow chart.

Of the 65 patients with COVID-19 who were initially identified during the study period, 13 were excluded: 7 because they could not undergo the tests due to clinical comorbidities; 2 who had recently had cataract surgery; 2 due to ocular comorbidity; 1 who was under 18 years of age; and 1 who did not agree to participate. An additional 7 patients who met the inclusion criteria were excluded because the data collected were not of sufficient quality for analysis. Therefore, the sample size at the study’s baseline (T0) was 45 COVID-19 patients. Over the study period, seven others were lost in the follow-up and did not contribute data at the second time point after 12 weeks (T1), leaving a final sample of 38 COVID patients who were included in the analysis (Figure 2). Their baseline characteristics are shown in Table 1. The mean age of the COVID-19 patient group was 57.3 years (standard deviation [SD] 13.6), and 50% (*n* = 19) were women. The mean interval from hospital admission to completion of the examinations was 3.1 days.

A sample of 30 non-COVID-19 patients was randomly selected from the historical database. Their mean age was 63.3 years, and 70% (*n* = 21) were women. In both groups, only one eye from each patient was included in the study in order to obtain independent observations. The right and left eyes were randomly selected in balanced groups. No statistically significant differences in terms of laterality were found between the two eyes of the same patient. The study of the right eye was included in 50% (*n* = 19) of the group of COVID patients and in 46.7% (*n* = 14) of the controls (Table 2).

Table 2 shows the cross-sectional analysis comparing the tomographic results of the COVID-19 patients at the time of hospital admission and with the historical non-COVID-19 controls. The only outcome showing significant differences was CRT, which was greater in the COVID-19 group. The rest of the variables also showed higher levels in patients with acute COVID-19, but these differences were not statistically significant.

Table 3 shows the results of the prospective longitudinal analysis of tomographic findings in COVID-19 patients during the acute (T0) versus late (T1) phase of the disease. None of the outcome variables showed statistically significant differences between time points, although the CRT values were slightly lower at T0 compared to T1. Regarding the presence of pathological findings in the posterior segment, none were observed in any of the participants at baseline or at the 12-week follow-up.

## 4. Discussion

The present study describes the tomographic status of the retina in unvaccinated patients during both the acute and recovery phase of COVID-19 and compares them with historical controls who had never been infected. Our results show that tomographic parameters in patients with COVID pneumonia do not change over the clinical course of the disease. The central retina seems to thicken and remain so for at least 12 weeks after infection. However, there was no evidence of inflammatory phenomena, hemorrhages, or subretinal or intraretinal fluid. No statistically significant changes were found in the central thickness of the choroid, ganglion cell layer, or retinal nerve fiber layer.

Previous OCT studies in COVID-19 patients were not conclusive, with notable disparities between authors. In a cross-sectional study in patients who had been previously admitted for complications derived from COVID-19, Beni et al. [14] found greater thickness of the peripapillary RNFL and lower density of the macular vessels compared with healthy eyes. Yildiz et al. [15] reported thickening in the outer nuclear layer and central fovea in patients who had recovered from the disease, while Burgos-Blasco et al. [16] observed greater thickness in the peripapillary RNFL in a population of children who had recovered from the disease compared to healthy controls. Seker et al. [17] also performed a cross-sectional study comparing patients who had recovered from the disease and healthy controls, identifying an increase in the thickness of the central RNFL and a thinning of the ganglion cell layer and internal nuclear layer in some patients. In contrast, Szkodny et al. [18] observed no significant changes in patients who had recovered from COVID-19 compared to healthy controls.

Investigations of patients during the acute phase of the disease are scarce. Bayram et al. [19] carried out a cross-sectional study in hospitalized patients with acute COVID-19, reporting a greater thickness of the external plexiform layer and the central choroid compared to people in the late phase of the disease and healthy controls. The participants of that study, the examination methods employed, and the study period were similar to those in our work, although a different OCT platform was used (Spectralis HRA OCT; Heidelberg Engineering, Heidelberg, Germany). However, our results did not corroborate the differences in the central choroidal thickness observed by Bayram et al. [19]. For their part, Oren et al. [20] described an increase in the central retinal thickness in patients in the early stages of recovery compared to healthy controls, along with thinning in the inner nuclear and ganglion cell layers.

Our results show a statistically significant difference in the central retinal thickness between patients with acute COVID-19 compared to historical controls. This may be due to the process of the swelling of the inner retinal tissues, stemming from an alteration in the function of the blood-retinal barrier, a cytopathic effect of SARS-CoV-2, or an alteration in the autoregulation of blood flow. In addition, no longitudinal differences were observed between the acute and recovery phases of the disease, suggesting that this thickening persists for at least 12 weeks after infection. Although the differences in the RNFL or GCL between acute COVID-19 patients and controls did not reach statistical significance, the COVID-19 group tended to present higher values, so the lack of statistical significance is likely affected by the small sample size. However, we did not find significant differences in CCT, suggesting that COVID-19 does not have a direct effect on the choroidal structure.

Furthermore, in our series there were no cases of acute ischemic or hemorrhagic findings in the retina. This result is of interest, as it contrasts with some previous reports. In May 2020, Marinho et al. [21] described hyperreflective lesions in the ganglion cell and inner plexiform layers of the retina in a series of six patients with a history of COVID-19 who underwent OCT studies. Moreover, Landecho et al. [22] observed cotton-wool exudates in 6 out of 27 patients with a history of hospital admission for COVID-19, suggesting that these findings may be due to a hypercoagulable state or a direct cytopathic effect of SARS-CoV-2 on the retina. Other authors disagree, indicating that these findings could be justified by a microangiopathic effect of SARS-CoV-2 in the retina [23,24]. Invernizzi et al. [25] described a greater frequency of hemorrhages, cotton-wool exudates, and vascular tortuosity in patients with COVID-19 compared to controls. Similarly, Patel et al. [26] reported in a retrospective study that hemorrhages and cotton-wool exudates were present in the retina of up to 30% of patients with COVID-19. The low prevalence of diabetes mellitus, hypertension, and smoking in our series of patients may be related to the absence of pathological findings, since these comorbidities are confounders independently associated with the appearance of ischemic, hemorrhagic, and inflammatory lesions in the retina.

To the best of our knowledge, this is the first study of its kind in unvaccinated patients in the acute phase of the disease, when contagion risks are highest. Indeed, performing the tests on the patients was very difficult given the risk to the research staff, the size of the OCT platform, the requirement for examination material, and the need to disinfect all equipment and instruments after examining each patient.

The study also has some limitations. First, a normative database of OCT imaging studies was used as the comparison group. Second, there could be unmeasured variables that could be associated with the response variable or that could be potentially confusing. Third, Lin et al. [27] suggested that the use of tropicamide prior to OCT imaging may increase the thickness of the retina and choroid in myopic patients. It is possible that this effect could be an explanation for the study’s findings, although it would be advisable to study this interaction in future research studies. Fourth, the period of data collection and field work was conditioned by strict hygiene and isolation measures that characterized the pandemic period before the introduction of SARS-CoV-2 vaccines. Once the vaccination protocols began, we decided not to include more patients due to the selection bias entailed in including patients who had received the vaccine, whose effects in the medium and long term were unknown. Therefore, all patients included in the study are unvaccinated patients with COVID pneumonia.

These circumstances help explain the low power of the study and its small size. A larger sample could have revealed stronger associations for variables such as RNFL and GCL. Furthermore, the lack of a control group with longitudinal follow-up precluded the implementation of a classic cohort design. The patient recruitment period was marked by a challenging healthcare context and low availability of screening tests, so we decided not to recruit a concurrent control group, since there was no guarantee that they would have been true healthy controls or simply patients with asymptomatic forms of COVID-19. Instead, we used a historical database of patients from the same area, with similar characteristics to the study group, and we performed the pertinent statistical analyses to ensure the comparability of the groups. Additionally, participants were people with COVID pneumonia who required hospital admission but not intensive care, as typical intensive care interventions such as orotracheal intubation and mechanical ventilatory support would have made it impossible to conduct the examinations. This selection of patients may imply a selection bias, because within the population of patients who required hospital admission due to the severity of their condition, those with the most severe disease could not be included. Examining such patients in the acute phase may have provided more evidence for our study, but performing OCT studies on these patients is impracticable with the current means. In addition, the follow-up time would not be sufficient to detect changes in retinal morphology. It would be useful to perform larger, multicenter studies with a longer follow-up in order to investigate tomographic differences in patients with COVID-19. However, the effect that vaccines might have on the results obtained is uncertain.

The increase in the central thickness of the retina might be due to a viral-induced inflammatory response of the retina. This increased thickness does not necessarily imply a clinical impact on patients, and, in the study patients, it was not related to pathologic changes, such as macular edema, neurosensory detachments, or retinal pigment epithelium detachments. However, as previous authors have suggested [15], this alteration might aggravate existing retinal diseases. Thus, these patients should be closely followed in case new pathologies emerge during the late recovery phase.

## 5. Conclusions

The results of this study show an increase in the central retinal thickness in patients with acute COVID pneumonia, which persists in the recovery phase. The rest of the clinical and tomographic variables evaluated showed no differences between patients in the acute phase of the disease and controls without COVID-19. In patients with COVID-19, the tomographic measures remained stable over both the acute and recovery phases.

## Figures and Tables

**Table 1:** Baseline characteristics in patients with COVID-19 (*n* = 38) during hospital admission and in non-COVID-19 controls (*n* = 30).

Baseline Variables	COVID-19 Group (*n* = 38)	Control Group (*n* = 30)
Age in years, mean (SD)	57.3 (13.6)	63.3 (15.7)
Women, *n* (%)	19 (50.0)	21 (70.0)
Tobacco use, *n* (%)	0 (0)	5 (16.7%)
Hypertension, *n* (%)	10 (26.3)	8 (26.7%)
Diabetes mellitus, *n* (%)	3 (7.9)	6 (20%)
Fibrinogen (mg/dL), mean (SD)	781 (224.6)	
D-dimers(μg/mL), mean (SD)	2.1 (3.5)	
IL-6 (pg/mL), mean (SD)	349 (496.2)	
Days from onset of symptoms to admission, mean (SD)	7.5 (4.1)	
Days from diagnosis to admission, mean (SD)	8.3 (4.1)	
Days from hospitalization to completion of eye exams, mean (SD)	3.1 (2.0)	
Posterior segment findings, *n* (%)	0 (0)	

SD: standard deviation.

**Table 2:** Cross-sectional analysis of tomographic findings in patients with acute COVID-19 versus non-COVID-19 controls.

Variables	Acute COVID-19 (*n* = 38)	Control Group (*n* = 30)	*p* Value
Age in years, mean (SD)	57.3 (13.6)	63.3 (15.7)	0.096
Women, *n* (%)	19 (50.0)	21 (70.0)	0.096
Eye laterality, right, *n* (%)	19 (50.0)	14 (46.7)	0.79
Central retinal thickness, µm, mean (SD)	264.5 (34.0)	246.6 (20.7)	0.006 *
Central choroidal thickness, µm, mean (SD)	237.0 (62.7)	227.1 (67.9)	0.54
Retinal nerve fiber layer, µm, median (IQR)	9.0 (7.0, 14.0)	7.0 (5.0, 12.0)	0.17
Ganglion cell layer, µm, median (IQR)	62.5 (52.0, 73.0)	57.0 (50.0, 66.0)	0.076

IQR: interquartile range; SD: standard deviation; * statistically significant difference.

**Table 3:** Prospective longitudinal analysis of tomographic outcomes in COVID-19 patients in the acute phase (T0) and at 12-week follow-up (T1).

Outcome Measure	Acute Phase	12-Week Follow-Up	*p* Value
Central retinal thickness, µm, mean (SD)	264.5 (34.0)	268.2 (34.3)	0.056 ^1^
Central choroidal thickness, µm, mean (SD)	237.0 (62.7)	240.6 (71.0)	0.99 ^1^
Retinal nerve fiber layer, µm, median (IQR)	9.0 (7.0, 14.0)	11.0 (8.0, 16.0)	0.21 ^2^
Ganglion cell layer, µm, median (IQR)	62.5 (52.0, 73.0)	66.0 (56.0, 76.0)	0.32 ^2^

IQR: interquartile range; SD: standard deviation. ^1^ General linear model of repeated measures; ^2^ Wilcoxon non-parametric test.

## Data Availability

The data presented in this study are available on request from the corresponding author. The data are not publicly available due to confidentiality reasons.
